# Pathogenic spectrum and risk factors of peritoneal dialysis-associated peritonitis: a single-center retrospective study

**DOI:** 10.1186/s12879-024-09334-9

**Published:** 2024-04-25

**Authors:** Linshuang You, Baoguo Zhang, Fan Zhang, Jianwen Wang

**Affiliations:** 1 Department of Nephropathy, The Central Hospital of Yongzhou, Yongzhou, China; 2 Department of Nephropathy, Third Xiangya Hospital of Central South University, Changsha, Hunan Province 410013 China; 3 Department of Critical Kidney Disease Research Center, Third Xiangya Hospital of Central South University, Changsha, China

**Keywords:** District, Peritoneal dialysis-associated peritonitis, Intractable peritonitis, Pathogenic spectrum, Risk factors

## Abstract

The present study aimed to explore the pathogenic spectrum and risk factors of peritoneal dialysis-associated peritonitis (Peritoneal dialysis associated peritonitis, PDAP) in Yongzhou, Hunan, China. The clinical and epidemiological data on regular peritoneal dialysis (Peritoneal dialysis, PD) between January 2016 and December 2020 in Yongzhou were collected for retrospective analysis. The related factors of peritonitis were evaluated by single-factor analysis, while risk factors of refractory PDAP were evaluated by multivariate logistic regression analysis.172/331 172 (51.9%) patients developed peritonitis. The risk factors of PDAP in PD patients included high C-reactive protein (C-reactive protein, CRP), low albumin(Albumin, ALB), low hemoglobin (Hemoglobin, Hb), low educational level (junior high school or lower), preference of spicy food, irregular diet, low annual household income, unfavorable fluid exchange conditions, unstable employment (including working as a farmer), and unfavorable humidity conditions (*P* < 0.05). 63/172 (36.6%) PDAP patients were intractable cases with a pathogenic bacteria positive rate of 74.60% in the peritoneal dialysate cultures, and 109/172 patients were non-intractable cases with a pathogenic bacteria positive rate of 53.21%. Gram-positive bacteria (G+) were detected in most of the dialysate cultures, with *Staphylococcus epidermidis* (*S. epidermidis*) as the most common type, while *Escherichia coli* (*E. coli*) was the most common Gram-negative bacteria (G-). Gram-positive bacteria were sensitive to vancomycin and linezolid, while G- bacteria were sensitive to imipenem and amikacin. Lifestyle, educational level, and environmental factors are the major contributors to PDAP in PD patients. Fungal and multi-bacterial infections are the major causes of death; PD is stopped for such patients.

**Supplementary Information:**

The online version contains supplementary material available at 10.1186/s12879-024-09334-9.

## Introduction

peritoneal dialysis is the most important alternative therapy for end-stage renal disease (ESRD) that protects remnant kidney function and can be easily administered at home [1, 2]. peritoneal dialysis-associated peritonitis can lead to peritoneal ultrafiltration and dialysis effects, thereby leading to complications that can result in PD failure and even death in patients [3–5].

peritoneal dialysis-associated peritonitis is defined according to Guidelines for the Prevention and Treatment of Peritoneal Dialysis Guidelines from International Association in 2022. After 5 days of appropriate antibiotic treatment, the PD fluid remains cloudy, or the white blood cell count in the permeated fluid continues to be > 0.1 × 10^9^/L [6].

Several factors can induce PDAP, and its pathogenesis is complicated. Some studies showed have shown that hypoalbuminemia is common in the maintenance of PD patients [7, 8]. The incidence of peritonitis in PD patients with hypoalbuminemia is increased significantly [9–12]. Based on the results of multiple observational studies (Dialysis outcomes and practice patterns study, DOPPS) analysis, persistent low potassium is also a risk factor for peritonitis [13–16].

Due to the widespread use of antibiotics in clinical practice, pathogenic bacteria spectrum and drug resistance of PDAP in PD centers are constantly changing, the resistance of pathogens to antibiotics shows an increasing trend, and the proportion of refractory PDAP increases year by year. In 2010, the International Society for Peritoneal Dialysis (International society for peritoneal dialysis, ISPD) emphasized the adjustment of medication according to the pathogen profile and treatment experience of PDAP in each dialysis center [17]. Yonzhou, a city in the southern part of Hunan, China, consists mostly of mountainous areas with an underdeveloped economy and insufficient access to the transport network. For patients with uremia in Yongzhou, PD is the major alternative therapy because frequent visits to the hospital in a week would be difficult. Based on the specific geographical location, environment, economy, and living habits, the present study analyzed the epidemiological data, pathogenic spectrum, drug sensitivity, and prognosis of PDAP patients in Yongzhou and explored the risk factors of PDAP in PD patients to provide suggestions for the prevention and management of the disease and evidence for the formulation of regional prevention, treatment strategies, and guidelines.

## Methods

### Study design and participants

The epidemiological and clinical data of 331 patients on regular PD in Yongzhou were collected between January 2016 and December 2020. All data were analyzed retrospectively. According to the International Society for Peritoneal Dialysis (ISPD) guidelines (2016) [18], PDAP can be diagnosed based on at least two of three criteria: (1) symptoms and signs, such as abdominal pain, cloudy dialysate, and/or fever; (2) white blood cell (White blood cell, WBC) count of ≥ 100 × 10^6^/L and multinucleated cell rate of ≥ 50% in the dialysate; (3) positive dialysate smear or culture. Intractable PDAP was diagnosed based on the criteria for PDAP and any one of the following: (1) no remission after five days of antibiotics and cloudy dialysate; (2) high WBC count after treatment; (3) fungal peritonitis; (4) complications with the PD catheter exit-site and tunnel infection; (5) new or recurrent peritonitis.

The inclusion criteria were as follows: (1) patients with > 3 months of experience in maintenance PD for uremia; (2) patients between 18- and 80-years-old; (3) patients on regular PD for at least 3 months; The exclusion criteria were as follows: (1) patients without complete clinical and follow-up records or participating in other clinical studies; (2) patients with fully recovered kidney function after PD; (3) patients producing bloody PD fluid; (4) patients producing chylous PD fluid; (5) patients with mental disorders and are unable to cooperate during treatment; (6) patients with comorbid acute or chronic blood system diseases; (7) patients with cerebrovascular accidents, such as cerebral infarction and hemorrhage; (8) patients with comorbid severe communicable diseases. Accordingly, all patients were assigned to the PDAP (Each patient with two or more PDAP cases was counted as one case, and the relevant data of the last peritonitis were selected) and non-PDAP groups. The PDAP group was further subdivided into intractable and non-intractable PDAP groups. This study was approved by the Ethics Committee of Yongzhou Central Hospital, and all patients signed the informed consent (No. 2,022,071,301).

### Sample collection

In sterile conditions, a volume of 10 mL dialysate (retained in the abdominal cavity for at least 4 h) was collected and placed in a blood culture bottle for testing using the Bact/ALERT 3D automated microbial detection system (Biomérieux, France). Then, the dialysate in the positive bottle was smeared and seeded on the Columbia blood agar and chocolate blood agar. The quality control bacteria included *E. coli*, (ATCC 25,922), *Staphylococcus aureus* (*S. aureus*, ATCC 25,923), and *Pseudomonas aeruginosa* (ATCC 27,853). The drug susceptibility test was conducted according to the method described by the Clinical and Laboratory Standards Institute (CLSI) (2016), using BACTEC™ 9000 (Becton Dickinson, USA), sterile paper discs (Oxiod, UK), and E-test plastic strips (Autobio, Zhengzhou, China).

### Cure and withdrawal criteria

According to ISPD guidelines, the cure criteria were as follows: (1) complete remission of PDAP signs and symptoms; (2) dialysate WBC count of < 100 × 10^6^/L and multinucleated cell rate < 50%, two consecutive negative cultures; (3) no recurrence after discontinuation of antibiotics. The withdrawal criteria [18, 19] were as follows: no alleviation within 2–3 weeks of antibacterial therapy, fungal peritonitis, intractable catheter exit-site and tunnel infection, multiple infections leading to PD catheter withdrawal, switching to hemodialysis, or death.

### Statistical analysis

SPSS 23.0 (IBM Corp.) was used for the data analysis. The enumeration data were expressed in percentage and ratio and compared using chi-square test. The measurement data were expressed as mean ± standard deviation (SD). Normally distributed data were subjected to *t*-test or one-way analysis of variance (ANOVA). For non-normal distribution and uneven variance, the data were subjected to rank-sum test. Factors in the univariate analysis were incorporated into the logistic regression model. *P* < 0.05 was considered statistically significant. Whonet 5.6 was used for the drug susceptibility test.

## Results

### Pathogenic spectrum

Among the 172 patients with PDAP, 63 (36.6%) were intractable cases. The pathogenic bacteria-positive rate in the PD cultures was 74.60% (47 cases). Gram-positive bacteria were detected in 24 cases (38.10%), among which *S. epidermidis* accounted for the highest proportion (9 cases, 14.29%). Gram-negative bacteria were detected in 18 cases (28.57%), and *E. coli* accounted for the highest proportion (8 cases, 12.70%). Furthermore, 2 (3.17%) cases presented multiple infections, and 3 (4.76%) had fungal infections. Moreover, 109 (63.37%) patients developed non-intractable PDAP. The peritoneal dialysate cultures of these patients presented a pathogenic bacteria-positive rate of 53.21% (58 cases). Among these cases, 32 (29.36%) were positive for G+, with 8 (7.34%) cases positive for *S. epidermidis*, while 26 (23.85%) were positive for G- with 8 (7.34%) cases positive for *E. coli* (Table 1).

**Table 1:** Pathogenic spectrum and prognosis of PDAP patients

	Non-intractable PDAP					Intractable PDAP			
	Number of cases	Number of cured	Number of transferred to hemodialysis	Number of deaths		Number of cases	Number of cured	Number of transferred to hemodialysis	Number of deaths
**Gram-positive bacteria**									
*Staphylococcus epidermidis*	8	7	1	0		9	6	3	0
*Staphylococcus xylosus*	2	1	1	0		1	0	1	0
*Human Staphylococcus subspecies*	3	2	1	0		1	1	0	0
*Enterococcus faecalis*	2	2	0	0		2	1	1	0
*Enterococcus faecium*	2	1	1	0		2	1	1	0
*Staphylococcus capitis*	3	3	0	0		2	2	0	0
*Staphylococcus palliative*	2	2	0	0		2	1	1	0
*Enterococcus gallinarum*	2	2	0	0		2	1	1	0
*Leuconostoc mesenteroides*	3	3	0	0		1	0	1	0
*Staphylococcus aureus*	2	2	0	0		1	1	0	0
*Staphylococcus haemolyticus*	3	3	0	0		1	1	0	0
**Gram-negative bacteria**									
*E. coli*	8	7	1	0		8	8	0	0
*Pseudomonas aeruginosa*	3	3	0	0		1	0	1	0
*Stenotrophomonas maltophilia*	3	3	0	0		1	1	0	0
*Aeromonas sobria*	2	2	0	0		2	1	1	0
*Achromobacter*	2	1	1	0		2	1	1	0
*Klebsiella pneumoniae*	3	3	0	0		2	2	0	0
*Enterobacter asburiae*	2	1	1	0		1	1	0	0
*Acinetobacter baumannii*	3	2	1	0		1	1	0	0
*Fungi infection*									
*Saccharomyces globoides*	0	0	0	0		2	0	1	1
*Candida Krusei*	0	0	0	0		1	0	1	0
Multiple infections	0	0	0	0		2	1	1	0
Negative results	51	49	2	0		16	14	2	0
Total	109	99	10	0		63	45	17	1

The drug resistance test results were similar for both intractable and non-intractable PDAP. Gram-positive bacteria detected from patients in both groups were sensitive to vancomycin and linezolid, but had a high resistance rate to penicillin G, oxacillin, clindamycin, cephazolin, and levofloxacin. Furthermore, G- were sensitive to imipenem and amikacin, but exhibited a high drug resistance rate for ceftazidime, gentamicin, ampicillin/sulbactam, piperacillin/tazobactam, and levofloxacin. Among the three fungal infection cases, one case was sensitive to all drugs, including flucytosine, fluconazole, voriconazole, amphotericin B, and itraconazole, while the other two cases were resistant to fluconazole, flucytosine, and voriconazole but were sensitive to the remaining drugs (Table 2).

**Table 2:** Drug resistance for G + and G- bacteria in PDAP patients

Antibiotics	Non-intractable PDAP						Intractable PDAP				
	Number of strains detected	Number of sensitive strains	Number of intermediately sensitive strains	Number of drug resistant strains	Drug resistance rate (%)		Number of strains detected	Number of sensitive strains	Number of intermediately sensitive strain	Number of drug resistant strains	Drug resistance rate (%)
**G+** **bacteria**											
Penicillin G	32	5	2	25	78.13		24	4	1	19	79.17
Oxacillin	32	4	1	27	84.38		24	3	2	19	79.17
Clindamycin	32	8	1	23	71.88		24	5	1	18	75.00
Vancomycin	32	32	0	0	0		24	24	0	0	0.00
Linzolid	32	32	0	0	0		24	23	0	1	4.17
Cephazolin	32	7	1	24	75.00		24	6	1	17	70.83
Levofloxacin	32	7	2	23	71.88		24	9	2	13	54.17
**G-** **bacteria**											
Ceftazidime	26	4	0	22	84.62		18	5	0	13	72.22
Ampicillin/sulbactam	26	5	0	21	80.77		18	4	0	14	77.78
Piperacillin/tazobactam	26	8	1	17	65.38		18	7	1	10	55.56
Gentamicin	26	6	1	19	73.08		18	5	1	12	66.67
Amikacin	26	26	0	0	0		18	17	0	1	5.56
Imipenem	26	26	0	0	0		18	18	0	0	0.00
Levofloxacin	26	3	0	23	88.46		18	4	1	13	72.22

Abbreviations: Gram-positive bacteria (G+) 、Gram-negative bacteria (G-)

### Analysis of risk factors for PDAP

The univariate analysis revealed that dialysis time, blood Hb, blood CRP, blood albumin (ALB), lactic dehydrogenase (Lactic dehydrogenase, LDH) in dialysate, occupation, educational level, income, diet preference, diet regularity, sanitation of fluid exchange conditions, and humidity, had a statistically significant impact on the occurrence of PDAP (*P* < 0.05, Tables 3 and 4). Furthermore, multivariate analysis revealed that high blood CRP [odds ratio (OR) = 12.354, 95% confidence interval (CI): 1.351–42.873)], low blood ALB (OR = 0.937, 95% CI 0.850–0.984), low blood Hb (OR = 0.924, 95% CI: 0.819–0.973), low educational level (junior high school or lower) (OR = 5.181, 95% CI: 1.514–15.379), preference of spicy food (OR = 4.563, 95% CI: 1.473–12.819), irregular diet (OR = 5.018, 95% CI: 1.419–11.328), low annual household income (OR = 4.133, 95% CI: 1.378–9.572), unfavorable fluid exchange conditions (OR = 3.572, 95% CI: 1.311–7.458), unstable employment (including working as a farmer) (OR = 4.933, 95% CI: 1.152–8.583), and unfavorable humidity conditions as, too high (OR = 2.951, 95%CI 1.257 ∼ 6.782) or too low (OR = 3.970, 95% CI: 1.182–5.637) were risk factors for PDAP in PD patients (*P* < 0.05, Table 5).

**Table 3:** Baseline characteristics for PDAP and control group (x ± s)

Factor	PDAP group (*n* = 172)	Control group (*n* = 159)	t	*P*
Age	52.95 ± 17.73	53.04 ± 17.35	0.047	0.963
Maintenance PD time (months)	26.84 ± 8.15	23.15 ± 9.17	3.875	< 0.001
Hospital visiting time (days)	6.05 ± 2.51	5.75 ± 1.97	1.203	0.230
WBC (×10^9^/L)	6.78 ± 2.74	6.91 ± 2.83	0.425	0.671
Hb (g/L)	89.74 ± 10.32	98.51 ± 13.74	6.597	< 0.001
TG (mmol/L)	1.64 ± 1.15	1.48 ± 0.98	1.357	0.176
BUN (mmol/L)	16.87 ± 6.58	15.97 ± 6.34	1.265	0.207
SCr (µmol/L)	751.46 ± 318.72	761.96 ± 305.89	0.305	0.760
CRP (mg/L)	125.73 ± 80.81	40.53 ± 16.74	13.037	< 0.001
ALB (g/L)	29.74 ± 6.12	35.04 ± 7.12	7.278	< 0.001
Dialysate WBC (×10^9^/L)	1.78 ± 1.43	1.68 ± 1.05	0.720	0.472
Dialysate ADA (U/L)	1.59 ± 1.48	1.54 ± 1.08	0.349	0.728
Dialysate LDH (U/L)	95.64 ± 71.82	68.97 ± 61.23	3.644	< 0.001
Dialysate TP (g/L)	2.60 ± 1.56	2.34 ± 1.28	1.183	0.238
Dialysate GLU (mmol/L)	19.43 ± 13.35	18.76 ± 14.23	0.442	0.659

Note: Hospital visiting time refers to the time between the appearance of peritonitis symptoms and the patient’s hospital visit; WBC: White blood cell count; Hb: Hemoglobin; TG: triglyceride; BUN: Blood urea nitrogen; SCr: serum creatinine; CRP: C-reactive protein; ALB: albumin; ADA: adenosine deaminase; LDH: lactate dehydrogenase; TP: total protein; GLU: Glucose; *P* < 0.05 indicated statistical significance

**Table 4:** Analysis of influencing factors of PDAP with chi-square test for PDAP in PD patients (*n*, %)

Factor		PDAP group (*n* = 172)	Control group (*n* = 159)	X^2^	*P*
Gender	Male	97 (56.40)	101 (63.52)	1.746	0.186
	Female	75 (43.60)	58 (36.48)		
Occupation	Unemployed	36 (20.93)	43 (27.05)	25.567	< 0.001
	Farmer	102 (59.30)	52 (32.70)		
	Office workers	18 (10.47)	34 (21.38)		
	Self-employed	16 (9.30)	30 (18.87)		
Annual household income	≥¥150,000 CNY	33 (19.19)	60 (37.74)	14.073	< 0.001
	<¥150,000 CNY	139 (80.81)	99 (62.26)		
Diet preference	Spicy and strong flavored food	138 (80.23)	62 (38.99)	58.758	< 0.001
	Light flavored food	34 (19.77)	97 (61.01)		
Diet regularity	Regular	85 (49.42)	102 (64.15)	14.805	< 0.001
	Irregular	87 (50.58)	57 (35.85)		
Fluid exchange conditions	Favorable	26 (15.12)	85 (53.46)	64.816	< 0.001
	Ordinary	78 (45.35)	57 (35.85)		
	Poor	68 (39.53)	17 (10.69)		
Educational level	Junior high school or lower	147 (85.47)	119 (74.84)	5.907	0.015
	Higher than junior high school	25 (14.53)	40 (25.16)		
Cause of kidney failure	Chronic glomerulonephritis	48 (27.91)	45 (28.30)	1.034	0.905
	Diabetic nephropathy	35 (20.35)	35 (22.01)		
	Obstructive nephropathy	40 (23.25)	39 (24.53)		
	Hypertensive nephropathy	32 (18.60)	29 (18.24)		
	Lupus nepheritis	6 (3.49)	3 (1.89)		
	Polycystic kidney	5 (2.91)	4 (2.51)		
	Gouty nephropathy	4 (2.33)	2 (1.26)		
	Vasculitis-associated nephritis	2 (1.16)	2 (1.26)		
Humidity	Favorable (40–70%)	23 (13.37)	98 (61.64)	19.219	< 0.001
	Too low (< 40%)	67 (38.95)	20 (12.57)		
	Too high (> 70%)	82 (47.68)	41 (25.79)		

Note: *P* < 0.05 denotes statistical significance

Abbreviations: CNY(ChinaYuan)

**Table 5:** Multivariate logistic regression analysis

Variable	*P*	OR	95% CI
High blood CRP	< 0.001	12.354	1.351–42.873
High blood ALB	0.032	0.937	0.850–0.984
High blood Hb	0.027	0.924	0.819–0.973
Low educational level (junior high school or lower)	0.009	5.181	1.514–15.379
Preference for spicy food	0.029	4.563	1.473–12.819
Irregular diet	0.012	5.018	1.419–11.328
Unstable employment (including working as a farmer)	0.017	4.933	1.152–8.583
Low annual household income	0.038	4.133	1.378–9.572
Unfavorable humidity (too low)	0.021	3.970	1.182–5.637
Unfavorable humidity (too high)	0.015	2.951	1.257–6.782
Poor fluid exchange conditions	0.019	3.572	1.311–7.458

Note: CRP: C-reactive protein; ALB: albumin; Hb: Hemoglobin;

Normal reference value range: CRP (0-10 mg/L) ALB (35–55 g/L) Hb (115–150 g/L)

### Outcomes of patients

Among the 63 patients with intractable PDAP, 45 were cured, and 18 stopped the PD (17 patients were transferred to hemodialysis after catheter removal, and one patient died). Patients with fungal and multiple infections had a poor prognosis. Among the 109 patients with non-intractable PDAP, 99 were cured, and 10 stopped the PD (all patients transferred to hemodialysis after catheter removal). Among the 159 patients who did not develop peritonitis, 6 patients stopped the PD (5 patients were transferred to hemodialysis after catheter removal, one patient received a kidney transplant), and no death occurred (Supplementary Table 1).

## Discussion

In the present study, PDAP occurred in 172/331 included patients, with an incidence (51.96%) significantly higher than that reported previously [20]. The findings indicated that PDAP should be under intensive focus and that other risk factors should also be explored to improve its prevention and treatment.

The study showed that the CRP levels in PDAP patients was significantly higher than the control group, which may serve as a predictive biomarker for the occurrence of peritonitis. C-reactive protein, a major indicator of inflammation, can activate the complement system to produce immune complexes, thereby damaging the endangium and indirectly affecting the pro-inflammatory factors. High CRP levels can be used to independently predict the severity of intractable peritonitis, which is consistent with previous findings [21–23]. Blood ALB is a critical parameter in evaluating nutritional status. Hypoalbuminemia is a common pathological condition of PD patients and is closely correlated to the host’s immune and inflammatory scenario. This can weaken a patient’s response and increase the risk of infection. Furthermore, hypoalbuminemia has been identified as a risk factor for the early occurrence of PDAP [24], which is consistent with the present findings, wherein low blood ALB was identified as a risk factor of PDAP and an independent risk factor for intractable PDAP. hemoglobin is also an indicator of nutrition in PD patients. In uremia patients, a decrease in hemopoietin can reduce hemopoiesis and cause renal anemia. Also, poor dietary intake would decrease the supply of nutrients for Hb synthesis, further aggravating anemia. Moreover, the pro-inflammatory factors are activated in the microinflammatory environment, thereby weakening host immunity and triggering inflammation. The present study revealed that a low Hb level is a risk factor for PDAP and an independent risk factor for intractable PDAP. Thus, the nutritional status should be monitored in PD patients to improve their condition and reduce the risk of PDAP [25–27].

In addition, a low education level (junior high school or lower), preference for spicy food, irregular diet, low annual household income, unfavorable fluid exchange conditions, unstable employment (including working as a farmer), and unfavorable humidity conditions (too high or too low) were risk factors of PDAP. These factors may represent the geological, climatic, educational, and lifestyle conditions in Yongzhou. An epidemiological investigation in Yongzhou identified the following features. First, Yongzhou is located in the south of Hunan, China, which is surrounded by mountains on three sides and occupied by low hills. This region has a mid-subtropical continental and monsoonal climate, which causes high humidity indoors, favoring the proliferation of bacteria. Second, Yongzhou is an underdeveloped region with a population that mainly comprises rural farmers who lack health education and bacteria-controlling knowledge. Third, the population in Yongzhou likes spicy and pickled foods. The long-term intake of these foods can distort the gut microinflammatory environment, and their irregular diet can also disrupt gastrointestinal function and may trigger peritonitis. Fourth, due to low household income, patients are often lost to follow-up, which delays the treatment of PDAP. Fifth, most of the patients raise poultry in their courtyards, which might pollute the fluid exchange environment. Based on these five factors, it can be inferred that doctors, patient’s family members, and local governments need to cooperate and propose a plan for the prevention and treatment of PDAP. The strategy should encompass health education, hygiene training, follow-up, fluid-exchange standardization, healthy diet, insurance coverage, environmental protection, and individualized therapy.

In the present study, the positive rate for intractable-PDAP-related bacteria was 74.60%, while the positive rate for non-intractable-PDAP-related bacteria was 53.21%; however, both were lower than that reported previously (78% and 81.52%, respectively) [28, 29]. This phenomenon could be explained by repeated infection or previous use of antibiotics before culture [30]. Furthermore, G + were the dominant pathogenic bacteria, which is in agreement with previous findings [31, 32]. The G + bacteria were represented by *S. epidermidis*, a coagulase-negative *Staphylococcus* that habitats on the skin. Its infection can induce the recurrence of PDAP. In addition, the high infection rate in the present study was consistent with that reported by previous studies [33, 34], indicating that the incorrect operation of fluid exchange remains the main factor for PDAP. G- bacteria were represented by *E. coli*, suggesting that PDAP is correlated with intestinal infection. Also, its infection rate is lower than that of *S. epidermidis* [35–37], indicating that PDAP is closely correlated with dirty food, constipation, diarrhea, and chronic enteritis.

According to ISPD guidelines [10], PD centers should use antibiotics with an antibacterial spectrum that covers G + and G- bacteria. The selection of drugs should be based on bacterial distribution and resistance. In the present study, the G + bacteria in PDAP patients were sensitive to vancomycin and linezolid, but resistant to cefazolin, while the G- bacteria were sensitive to imipenem and amikacin, but resistant to ceftazidime and gentamicin. Therefore, for PDAP patients in Yongzhou, vancomycin (or linezolid) and imipenem (or amikacin) were recommended before reporting the chemosensitivity assay results or after the completion of the bacterial culture.

With the widespread use of antimicrobial drugs, there has been a gradual increase in the prevalence of resistant strains. The initial treatment regimen adopted by our institution during this period consisted of first-generation cephalosporins (cefazolin) combined with third-generation cephalosporins (cefotaxime), resulting in relatively high rates of resistance to these antibiotics. Recent studies [38–40] have shown an upward trend in resistance rates to commonly used antibiotics such as cefazolin, ampicillin, and cefotaxime. Additionally, research indicates that Gram-positive pathogens may develop resistance to almost all clinically available antimicrobial drugs, making them more likely to produce multidrug-resistant (MDR) strains compared to Gram-negative bacteria [41]. According to the 2022 ISPD guidelines [42], antibiotic selection should be based on the specific circumstances of the healthcare institution and should cover both Gram-positive and Gram-negative bacteria. For Gram-positive bacterial infections, first-generation cephalosporins or vancomycin are recommended, while for Gram-negative bacterial infections, third-generation cephalosporins or aminoglycosides are preferred. It is imperative for us to adhere to, but not blindly follow, the ISPD guidelines, and regularly assess the spectrum of hospital pathogens and initial treatment regimens to minimize the emergence of resistant strains.

In the present study, two cases with multiple infection were tested for G-, which may have originated from enteral infection. The treatment had poor efficacy, and the infection recurred repeatedly. Finally, PD was replaced by hemodialysis. Three cases presented fungal infection, which included two cases of *Torulopsis glabrata* (*T. glabrata*) and one case of *Candida Krusei*. The case infected with *T. glabrata* was switched to antifungal therapy but died. This finding suggested that fungal infections or multiple infections are the main causes of death or withdrawal from PD. For these patients, studies recommended that the catheter should be withdrawn earlier [43, 44]. Moreover, the peritoneal function should be retained, and systemic infection should be prevented.

For refractory peritonitis, we need to identify relevant factors and administer targeted treatments for the underlying causes. Multidrug resistance poses a high risk for refractory and recurrent peritonitis. Refractory peritonitis is a major reason for peritoneal dialysis (PD) patients withdrawal, potentially leading to residual infections affecting PD re-initiation and reducing technique survival rates [45]. Recurrent peritonitis increases the risk of further recurrence and relapse. Given the high prevalence of multidrug-resistant infections in peritoneal dialysis-associated peritonitis (PDAP) patients, it is crucial to promptly identify and control their risk factors, minimize the occurrence of multidrug infections, and initiate appropriate antibiotic therapy based on pathogen distribution and resistance characteristics. This is essential for reducing adverse clinical outcomes such as refractory and recurrent peritonitis.

## Conclusions

In summary, This study retrospectively analyzed the clinical and epidemiological data of peritoneal dialysis (PD) patients in Yongzhou City from January 2016 to December 2020.Ultimately, it was found that for PDAP patients, G + bacteria were sensitive to vancomycin and linezolid, while G- bacteria were sensitive to imipenem and amikacin. Lifestyle, educational level, and environmental factors are the major contributors to PDAP in PD patients. Fungal and multi-bacterial infections are the major causes of death; PD is stopped for such patients.

### Study limitation

The present retrospective study was based on the data obtained from a single center in Yongzhou, thus lacking data from multiple centers. Presently, the investigators are establishing a PD database of patients from other areas. A multicenter and prospective study would be performed based on this database to provide evidence for PDAP prevention and treatment.

### Electronic supplementary material

Below is the link to the electronic supplementary material.

Supplementary Material 1

## Data Availability

All data generated or used during the study are available from the corresponding author by request.
