# Supplementary material for: Pathogenic spectrum and risk factors of peritoneal dialysis-associated peritonitis: a single-center retrospective study
Source: BMC Infect Dis. 2024 Apr 25;24:440. doi: 10.1186/s12879-024-09334-9 (PMC11044422; doi:10.1186/s12879-024-09334-9)
Supplement: Supplementary file 1 — Supplementary Material 1 [file 12879_2024_9334_MOESM1_ESM.doc]

**Supplementary Table 1. Outcomes of PDAP patients**

| Disease | Number | Cure | Catheter withdrawal | Death | Kidney transplantation | Cure rate (%) | Dropout rate (%) |
| --- | --- | --- | --- | --- | --- | --- | --- |
| Intractable PDAP (*n*) | 63 | 45 | 17 | 1 | 0 | 71.43 | 28.57 |
| Non-intractable PDAP (*n*) | 109 | 99 | 10 | 0 | 0 | 90.83 | 9.17 |
| Non-PDAP (*n*) | 159 | - | 5 | 0 | 1 | - | 3.77 |
| Total (*n*) | 331 | - | 32 | 1 | 1 | - | 10.27 |
